# Selective PPARγ modulator diosmin improves insulin sensitivity and promotes browning of white fat

**DOI:** 10.1016/j.jbc.2023.103059

**Published:** 2023-02-24

**Authors:** Jian Yu, Yepeng Hu, Maozheng Sheng, Mingyuan Gao, Wenxiu Guo, Zhe Zhang, Dongmei Wang, Xia Wu, Jin Li, Yantao Chen, Wenjun Zhao, Caizhi Liu, Xiangdi Cui, Xin Chen, Cheng Zhao, Huang Chen, Junjie Xiao, Shijie Chen, Cheng Luo, Lingyan Xu, Xuejiang Gu, Xinran Ma

**Affiliations:** 1 Department of Endocrine and Metabolic Diseases, The First Affiliated Hospital of Wenzhou Medical University, Wenzhou, Zhejiang, China; 2 Shanghai Key Laboratory of Regulatory Biology, Institute of Biomedical Sciences and School of Life Sciences, East China Normal University, Shanghai, China; 3 Joint Center for Translational Medicine, Fengxian District Central Hospital, Shanghai, China; 4 Cardiac Regeneration and Ageing Lab, Institute of Cardiovascular Sciences, School of Life Sciences, Shanghai University, Shanghai, China; 5 State Key Laboratory of Drug Research, Drug Discovery and Design Center, The Center for Chemical Biology, Shanghai Institute of Materia Medica, Chinese Academy of Sciences, Shanghai, China; 6 Chongqing Key Laboratory of Precision Optics, Chongqing Institute of East China Normal University, Chongqing, China

**Keywords:** diosmin, selective PPARγ modulator, PPARγ phosphorylation, insulin sensitivity, browning of white fat tissue

## Abstract

Peroxisome proliferator–activated receptor γ (PPARγ) is a master regulator of adipocyte differentiation, glucolipid metabolism, and inflammation. Thiazolidinediones are PPARγ full agonists with potent insulin-sensitizing effects, whereas their oral usage is restricted because of unwanted side effects, including obesity and cardiovascular risks. Here, *via* virtual screening, microscale thermophoresis analysis, and molecular confirmation, we demonstrate that diosmin, a natural compound of wide and long-term clinical use, is a selective PPARγ modulator that binds to PPARγ and blocks PPARγ phosphorylation with weak transcriptional activity. Local diosmin administration in subcutaneous fat (inguinal white adipose tissue [iWAT]) improved insulin sensitivity and attenuated obesity *via* enhancing browning of white fat and energy expenditure. Besides, diosmin ameliorated inflammation in WAT and liver and reduced hepatic steatosis. Of note, we determined that iWAT local administration of diosmin did not exhibit obvious side effects. Taken together, the present study demonstrated that iWAT local delivery of diosmin protected mice from diet-induced insulin resistance, obesity, and fatty liver by blocking PPARγ phosphorylation, without apparent side effects, making it a potential therapeutic agent for the treatment of metabolic diseases.

The prevalence of type 2 diabetes has increased considerably worldwide in recent decades, accompanied with increased incidences of associated comorbidities and growing trends of morbidity and mortality, posting increasing economic burden and social disadvantages (1, 2). Although the frontline diabetic drugs including GLP-1 analog, SLGT2 inhibitor, and metformin were widely used, natural compounds and their derivations that have potential use as therapeutics or daily supplements with glycemic control effects are still in high demands.

Adipose tissues play critical roles in glucose and lipid homeostasis (3). Adipose tissues have been divided into three types: brown, beige, and white adipose tissues, according to their location, morphology, and function (4, 5). Excess accumulation of white adi2pose tissue can induce detrimental metabolic effects, whereas beige and brown adipose tissues have the potential to improve metabolism (6). The beige (also known as “brown-like”) adipose tissue mainly exists in the subcutaneous fat pads with inducible thermogenic capacity in response to stimulations like cold or β3 agonists (7). In addition to sympathetic nervous systems and β-adrenergic signaling, beige adipocytes could also be activated in a cell-autonomous manner (8). Beige adipocyte activation increases energy expenditure and thermogenesis, as well as functions as a metabolic sink to consume excess lipid and glucose, thus reduces obesity and hyperlipidemia and improves insulin sensitivity (9), emphasizing beige fat as an attractive target for obesity and diabetes treatments.

Peroxisome proliferator–activated receptor γ (PPARγ) is a member of the nuclear receptor family of transcription factors, which governs adipocyte differentiation and thermogenesis, insulin sensitivity, and inflammation inhibition (10, 11, 12). Classic PPARγ-full agonist thiazolidinediones (TZDs) could activate PPARγ and increase insulin sensitivity of peripheral tissues (fat tissue, skeletal muscle, liver, etc.) to achieve a hypoglycemic effect (13). However, their usage has been limited in clinical treatment because of unwanted side effects, such as weight gain, cardiovascular risk, edema, and osteoporosis (14). Thus, selective PPARγ modulators acting through alternative mechanisms to modulate PPARγ activity without activating the full transcriptional program were developed (15). Particularly, it has been found that high-fat feeding activates the protein kinase cyclin-dependent kinase 5 (Cdk5) resulting in phosphorylation of PPARγ at Ser273. Compounds blocking PPARγ phosphorylation at Ser273 would reverse a specific set of diabetic gene programs and exhibit antidiabetic effects (16). Recently, studies demonstrated that dephosphorylation of PPARγ at Ser273 with PPARγ partial agonist or nonagonist PPARγ ligands including SR1664, MRL24, GQ-16, and CDK5 inhibitor roscovitine conveyed insulin-sensitizing and/or antiobesity effects, whereas at the same time avoided undesirable adverse effects of TZDs (17, 18, 19). These observations indicate that blocking phosphorylation of PPARγ Ser273 represents a novel avenue of drug development for diabetes and obesity treatments.

Diosmin is a flavone glycoside, a natural compound extracted from dehydrated pericarps of different citrus fruits. Diosmin is a safe and nontoxic clinical medication regularly used for treating venous disease, that is, chronic venous insufficiency and acute or chronic hemorrhoidal disease (20, 21). Mechanistically, diosmin exerted its protective role on venous system by prolonging the vasoconstriction effects of β-adrenergic signaling and decreasing capillary permeability (22). Diosmin also exhibited anti-inflammatory, antioxidation activities, and anticancer through its regulation on NF-κB signaling pathway and phosphatidylinositol 3-kinase/Akt pathway (23, 24, 25). Recently, in addition to its function in vascular protection and inflammation, metabolic implications of diosmin were documented as it shows liver protective function by improving bile duct ligation–induced liver defects and promote glucose homeostasis in models of type I diabetes (26, 27).

In the present study, computer virtual screening was performed in a natural small-molecule compound library to screen for compounds with potent binding to PPARγ. We identified diosmin that characterized binding affinity with PPARγ and blocked its S273 site. Our results demonstrated that diosmin treatment induced antidiabetic effects in beige fat and adipocytes without affecting adipogenesis. Moreover, local administration of compounds in adipose tissues has attracted great attentions for enhanced treatment efficiency and minimized side effects. For example, it has been shown that local administration of nanoparticle-formulated rosiglitazone in subcutaneous fat (inguinal white adipose tissue [iWAT]) induces adipose tissue browning and prevents obesity (28). Besides, we have developed a highly efficient fluoropolypeptide for the delivery of siRNA-based therapeutics directly into iWAT to combat obesity and metabolic diseases (29). In addition, we and others have recently shown that local hyperthermia therapy on beige fat with photothermal nanoparticles induces thermogenesis to combat obesity without obvious systemic side effects (30, 31). Considering these previous studies, in the present study, we selected local injection of diosmin into iWAT and found that diosmin reduced obesity and hepatic steatosis under high-fat diet (HFD) *via* browning of white fat and enhanced energy expenditure without obvious side effects. These results indicated that diosmin is a potential agent for the treatment of obesity and diabetes.

## Results

### Diosmin is a selective PPARγ modulator and enhances glucose uptake

**Figure 1:**
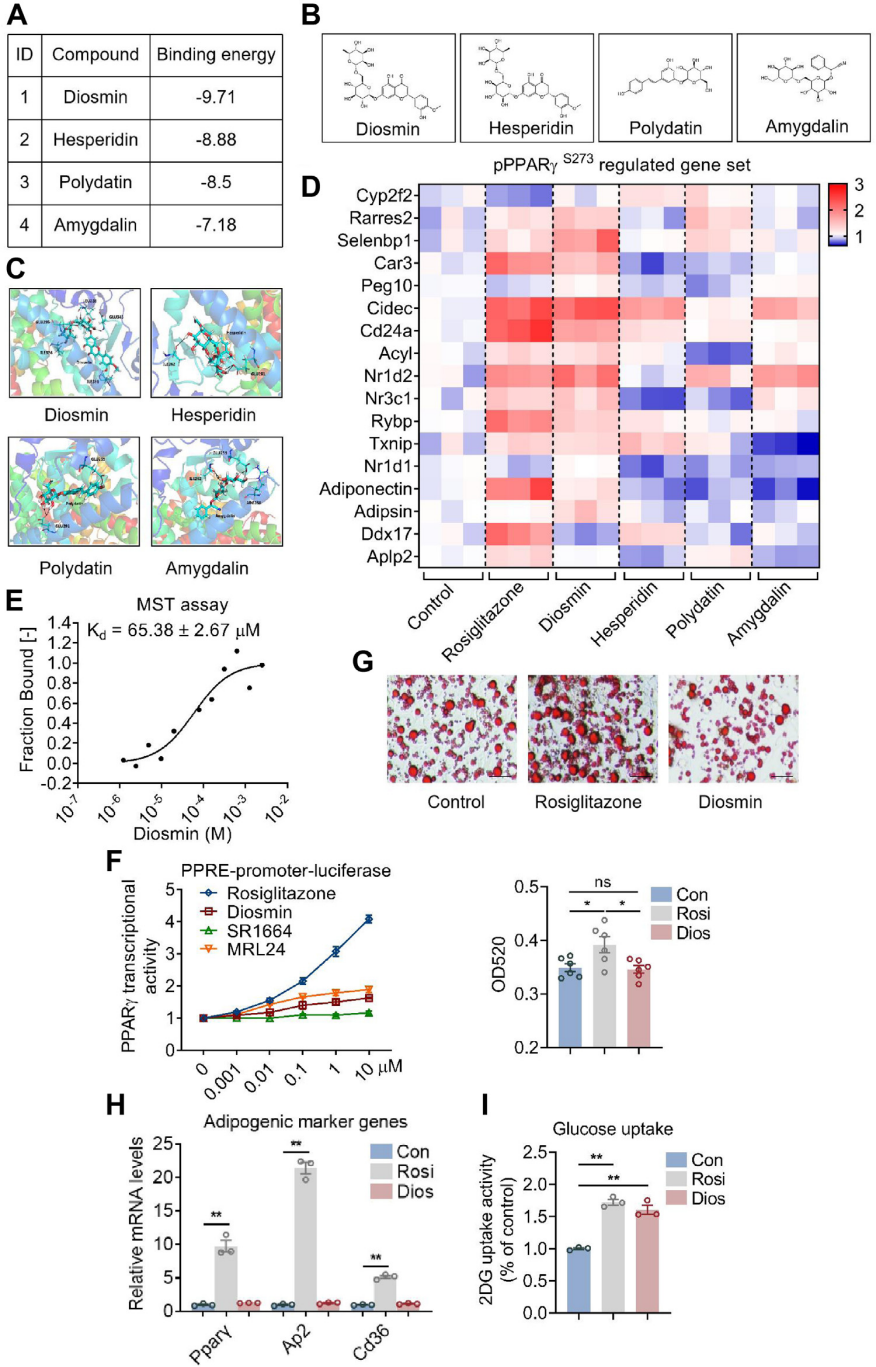
**Diosmin (Dios) blocks PPARγ phosphorylation in beige adipocytes.***A*, binding free energy, (*B*) chemical structure, and (*C*) molecular docking model of Dios, hesperidin, polydatin, and amygdalin. *D*, expression of gene sets regulated by PPARγ S273 phosphorylation in beige adipocytes treated with control, rosiglitazone (Rosi), Dios, hesperidin, polydatin, and amygdalin (n = 3). *E*, microscale thermophoresis assay to assess the binding of PPARγ and Dios. *F*, transcriptional activity of a PPARγ-responsive element (PPRE) after treatment with Rosi or Dios (n = 3). *G*, representative images and quantity of Oil Red staining and (*H*) expression of adipogenic marker genes of beige adipocytes treated with control (Con), Rosi, or Dios (n = 3). The scale bar represents 50 μm. *I*, glucose uptake in beige adipocytes treated with Con, Rosi, or Dios (n = 3). Data are presented as mean ± SEM and ∗*p* < 0.05, ∗∗*p* < 0.01 compared with control group. PPARγ, peroxisome proliferator–activated receptor γ.

To identify novel PPARγ ligand that blocks PPARγ pS273, computer virtual screening was performed using molecular docking strategy with 3000 natural small-molecule compounds. Among the positive candidates, four compounds including diosmin, hesperidin, polydatin, and amygdalin featured highest absolute binding free energy. Among them, diosmin piqued our interest as it features strongest binding affinity, whereas molecular docking studies predicted its binding pocket nearing PPARγ S273 (Fig. 1, *A*–*C*). Indeed, compared with other three compounds, diosmin increased a specific set of diabetic genes dysregulated in the scenario of CDK5-induced PPARγ S273 phosphorylation in a similar pattern as the PPARγ full agonist rosiglitazone did (16) (Fig. 1*D*).

We further conducted microscale thermophoresis (MST) experiment, which confirmed interactions between PPARγ and diosmin, indicated its potential usage for diabetic control as a lead compound (Fig. 1*E*). Of note, compared with rosiglitazone, diosmin showed weak transcriptional activity in a PPARγ-responsive element–driven luciferase assay to the similar extent of the reported PPARγ nonagonist SR1664 and partial agonist MRL24 (16, 17) (Fig. 1*F*). Besides, in contrast to rosiglitazone, we found that diosmin has minor effects on adipocyte differentiation as shown by Oil Red staining and classic adipogenic marker gene expression (Fig. 1, *G* and *H*). *Via* 2-deoxyglucose (2-DG) uptake assay, we showed that diosmin significantly increased 2-DG uptake upon insulin treatment in beige adipocytes, suggesting its binding to PPARγ for enhanced glucose utilization (Fig. 1*I*). Overall, these data suggest that diosmin functions as a PPARγ selective modulator to enhance glucose uptake in beige adipocytes.

### Diosmin blocks PPARγ phosphorylation and improves diabetic gene programs both *in vitro* and *in vivo*

**Figure 2:**
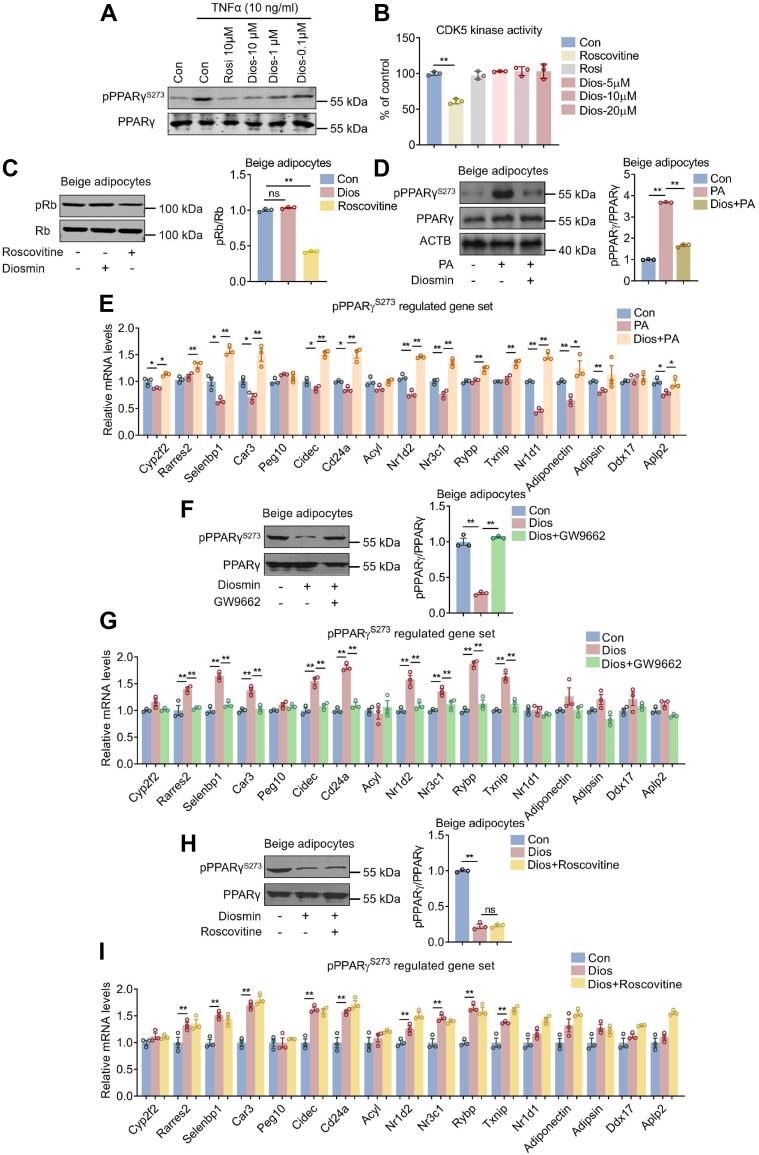
**Diosmin is a selective PPARγ modulator.***A*, TNF-α induced phosphorylation of PPARγ S273 in beige adipocytes treated with rosiglitazone or diosmin at indicated doses. *B*, CDK5 activity assay in beige adipocytes treated with control, diosmin, rosiglitazone, or roscovitine (n = 3). *C*, protein levels of p-Rb and Rb in beige adipocytes treated with control, diosmin, or roscovitine (n = 3). *D*, protein levels of p-PPARγ (S273) and PPARγ and (*E*) expression of gene sets regulated by PPARγ S273 phosphorylation in beige adipocytes treated with control, PA, or diosmin + PA (n = 3). *F*, protein levels of p-PPARγ (S273) and PPARγ and (*G*) expression of gene sets regulated by PPARγ S273 phosphorylation in beige adipocytes treated with control, diosmin, or diosmin + GW9662 (n = 3). *H*, protein levels of p-PPARγ (S273) and PPARγ and (*I*) expression of gene sets regulated by PPARγ S273 phosphorylation in beige adipocytes treated with control, diosmin, or diosmin + roscovitine (n = 3). Data are presented as mean ± SEM and ∗*p* < 0.05, ∗∗*p* < 0.01 compared with control group. PPARγ, peroxisome proliferator–activated receptor γ.

Since diosmin has been predicted to bind near the PPARγ S273 site and that we have shown diosmin activates the diabetic gene programs regulated by PPARγ pS273 as potently as rosiglitazone (Fig. 1*D*), we then examined whether diosmin exerts its modulator functions by blocking PPARγ S273 phosphorylation. Tumor necrosis factor alpha (TNFα) has been reported to induce PPARγ S273 phosphorylation (16). Of note, similar to rosiglitazone, diosmin inhibited TNFα-induced PPARγ pS273 in a dose-dependent manner in beige adipocytes (Fig. 2*A*). Of note, both rosiglitazone and diosmin treatment showed no obvious effect on CDK5 activity, with CDK5 inhibitor roscovitine as a positive control (Fig. 2*B*). Besides, we found that diosmin addition had no effect on the phosphorylation of Rb protein, another well-characterized CDK5 substrate (Fig. 2*C*), indicating diosmin may not impact PPARγ phosphorylation through its regulation on CDK5 activity. Moreover, consistent with previous results (32), palmitic acid induced PPARγ S273 hyperphosphorylation and worsened previously reported PPARγ pS273-regulated diabetic gene programs in beige adipocytes (16, 17, 33, 34), which were significantly reversed by diosmin treatment (Fig. 2, *D* and *E*). Furthermore, diosmin administration decreased PPARγ S273 phosphorylation and improved diabetic gene programs in beige adipocytes, whereas these effects were lost in adipocytes treated with PPARγ antagonist GW9662, possibly because of the confirmational change of PPARγ upon GW9662 treatment (Fig. 2, *F* and *G*). Roscovitine is a specific inhibitor of CDK5, the kinase responsible for PPARγ S273 phosphorylation (19). Interestingly, compared with diosmin administration alone, combined treatment of diosmin and roscovitine failed to further reduce PPARγ S273 phosphorylation and modulate diabetic gene programs (Fig. 2, *H* and *I*), indicating that PPARγ pS273 is indispensable for diosmin functionality. Overall, these data showed that diosmin modulates diabetic gene programs *via* inhibition of PPARγ S273 phosphorylation *in vitro*.

**Figure 3:**
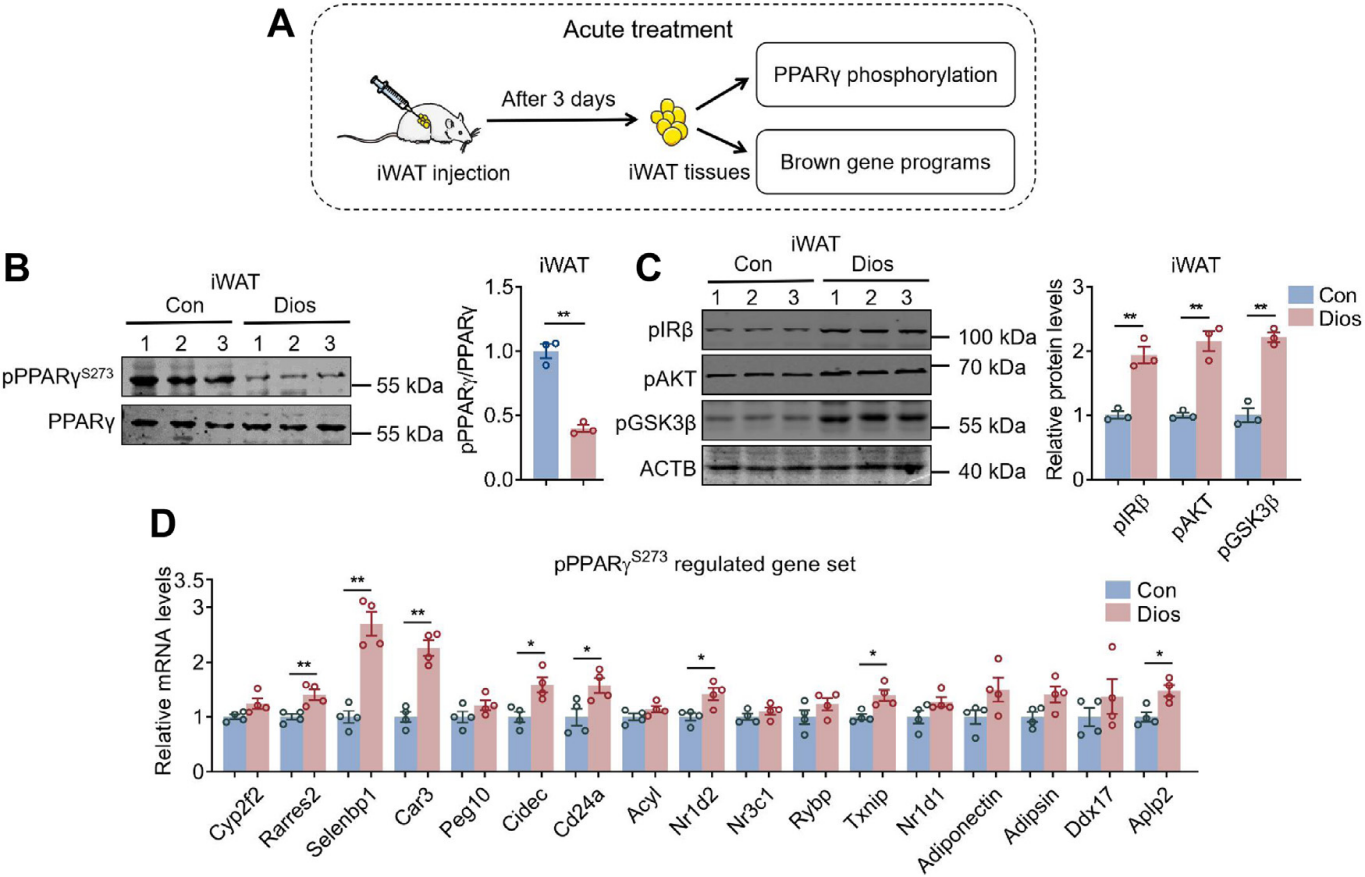
**Acute diosmin (Dios) administration improves diabetic gene programs in iWAT of mice.***A*, experimental model of acute control (Con) or Dios administration in mice with iWAT unilateral injection (n = 4). *B*, protein levels of S273 p-PPARγ, (*C*) p-IRβ, p-AKT, and p-GSK3β, (*D*) expression of gene set regulated by PPARγ S273 phosphorylation in iWAT of mice after acute Dios administration. Data are presented as mean ± SEM and ∗*p* < 0.05, ∗∗*p* < 0.01 compared with control group. iWAT, inguinal white adipose tissue; PPARγ, peroxisome proliferator–activated receptor γ.

We then set out to unravel the effects of diosmin administration *in vivo*. For acute gene expression analysis, control, diosmin, rosiglitazone, hesperidin, polydatin, and amygdalin were injected unilaterally into inguinal fat pads of wildtype C57BL/6 mice (Fig. 3*A*). After 3 days, we found that diosmin decreased PPARγ S273 phosphorylation levels, elevated insulin signaling pathway including p-IRβ, p-AKT, and p-GSK3β, as well as improved PPARγ pS273-related diabetic genes in iWAT, similar to rosiglitazone (Fig. 3, *B*–*D*), suggesting that diosmin modulated PPARγ phosphorylation, insulin signaling, and diabetic gene programs *in vivo*. Interestingly, we found that hesperidin, polydatin, and amygdalin exhibited significantly weaker transcriptional activity compared with the full agonist rosiglitazone (Fig. S1), wheres they showed minimal effects on PPARγ phosphorylation and insulin sensitivity improvement *in vivo* (Fig. S2).

### Diosmin induced brown gene programs both *in vitro* and *in vivo*

**Figure 4:**
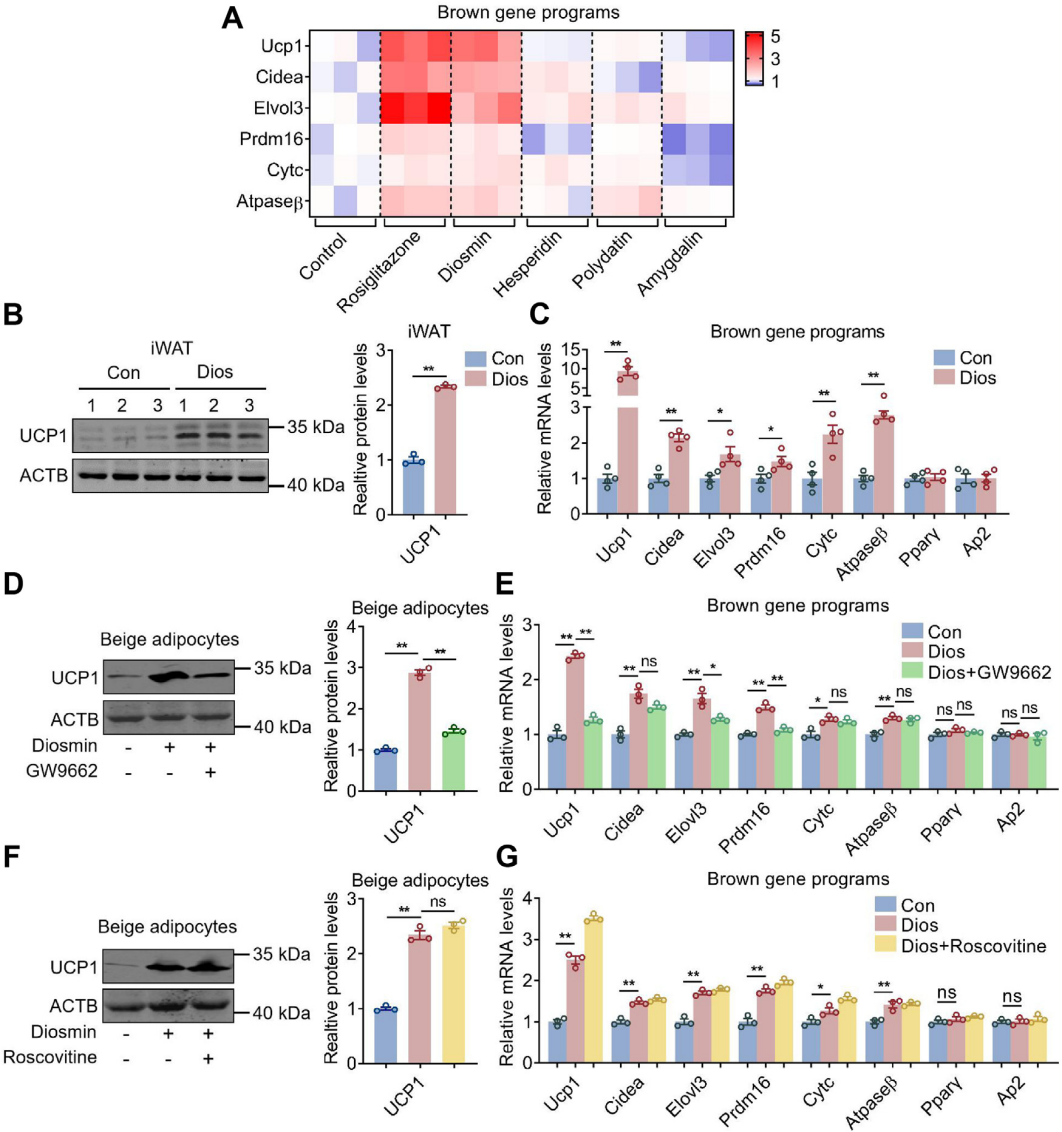
**Diosmin administration induces browning gene programs both *in vitro* and *in vivo*.***A*, heatmap of expression levels of brown gene programs in beige adipocytes treated with control, rosiglitazone, diosmin, hesperidin, polydatin, or amygdalin (n = 3). *B*, uncoupling protein 1 (UCP1) protein levels and (*C*) expression levels of brown gene programs in inguinal white adipose tissue (iWAT) of mice after acute diosmin administration (n = 4). *D*, UCP1 protein levels and (*E*) expression levels of brown gene programs in beige adipocytes treated with control, diosmin, or diosmin + GW9662 (n = 3). *F*, UCP1 protein levels and (*G*) expression levels of brown gene programs in beige adipocytes treated with control, diosmin, or diosmin + roscovitine (n = 3). Data are presented as mean ± SEM and ∗*p* < 0.05, ∗∗*p* < 0.01 compared with control group.

In addition to diabetic gene programs, we found that diosmin also increased brown gene programs in beige adipocytes in a similar pattern as rosiglitazone did, which was also recapitulated in iWAT of mice after diosmin administration acutely, in addition to increased protein levels of uncoupling protein 1 (UCP1) (Fig. 4, *A*–*C*). Moreover, increased protein levels of UCP1 and mRNA levels of brown gene programs after diosmin treatment were blunted in adipocytes treated with PPARγ antagonist GW9662 (Fig. 4, *D* and *E*). Meanwhile, sole diosmin treatment promoted UCP1 protein level and the expression of brown gene programs, whereas combined usage of diosmin and CDK5 inhibitor roscovitine showed no further effects (Fig. 4, *F* and *G*).

Moreover, we also examined the effects of diosmin on insulin sensitivity and thermogenic gene programs in mice *via* acute oral delivery, using rosiglitazone as a positive control. Indeed, rosiglitazone and diosmin both significantly promoted insulin sensitivity in mice iWAT, including decreased PPARγ S273 phosphorylation, improved diabetic genes dysregulated by PPARγ pS273, and elevated pIRβ, pAKT, and pGSK3β levels (Fig. S3, *A*–*D*), as well as increased brown gene programs and UCP1 protein levels (Fig. S3, *E* and *F*) in iWAT. Notably, oral administration of rosiglitazone significantly increased adipogenic gene expression in iWAT, which was absent upon diosmin administration (Fig. S3*E*). These data suggest that diosmin may exhibit a potential insulin-sensitizing and white fat browning effects in mice.

### Diosmin ameliorated insulin resistance and obesity in mice under HFD

**Figure 5:**
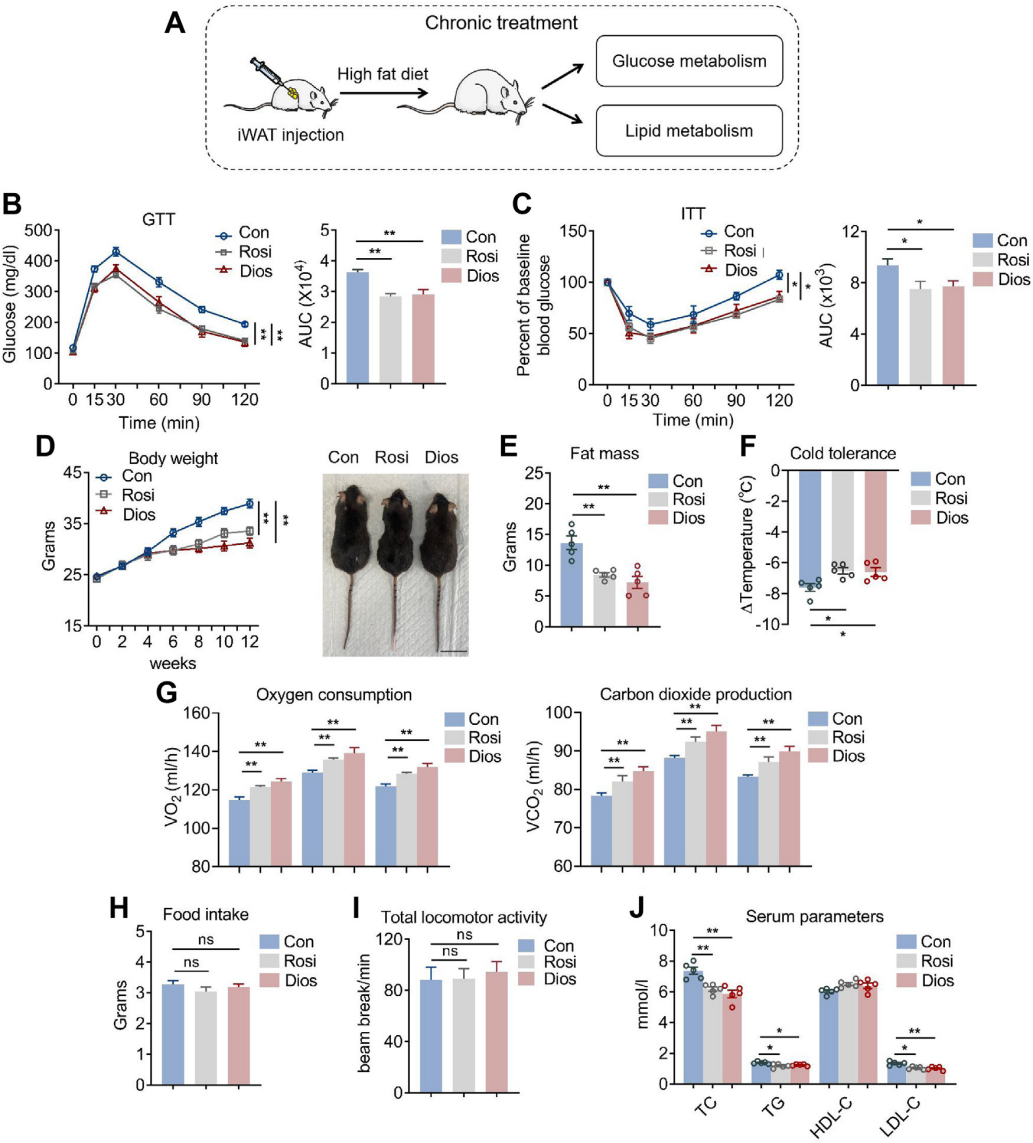
**Diosmin (Dios) improves insulin sensitivity and promotes browning of white fat in mice under high-fat diet (HFD).***A*–*J*, metabolic performances of HFD mice treated with control (Con), rosiglitazone (Rosi), or Dios (n = 5). *A*, experimental model of chronic control, Rosi, or Dios administration in mice with bilateral inguinal white adipose tissue (iWAT) injection. *B*, glucose tolerance test (GTT) and area under the curve (AUC). *C*, insulin tolerance test (ITT) and AUC. *D*, body weight and mice appearance; The scale bar represents 2 cm. *E*, fat mass. *F*, rectal temperature changes of mice during 5 h cold exposure. *G*, energy expenditure as shown by oxygen consumption and carbon dioxide production. *H*, total locomotor activity. *I*, food intake. *J*, analysis of serum parameters. Data are presented as mean ± SEM and ∗*p* < 0.05, ∗∗*p* < 0.01 compared with control group.

We thus examined whether diosmin administration chronically could ameliorate insulin resistance and obesity in mice under HFD. Indeed, similar to rosiglitazone, 12-week diosmin intervention improved insulin sensitivity in mice as shown by lower fasting and random glucose levels, better performances in glucose and insulin tolerance tests, as well as decreased body weights and fat mass compared with control group (Figs. 5, *A*–*E* and S4). These phenotypes were accompanied with enhanced thermogenic capacity and energy expenditure as demonstrated by increased core temperature during cold exposure (Fig. 5*F*) as well as enhanced oxygen consumption and carbon dioxide production in diosmin-treated group compared with control groups, without obvious changes in locomotor activity and food intake (Fig. 5, *G*–*I*). Furthermore, serum analysis showed that diosmin decreased serum total cholesterol, total triglyceride, and low-density lipoprotein–cholesterol levels (Fig. 5*J*). These data suggested that diosmin treatment improved insulin sensitivity, alleviated obesity, and hyperlipidemia in mice under HFD.

### Diosmin ameliorated adiposity and hepatic steatosis with reduced inflammation in HFD mice

**Figure 6:**
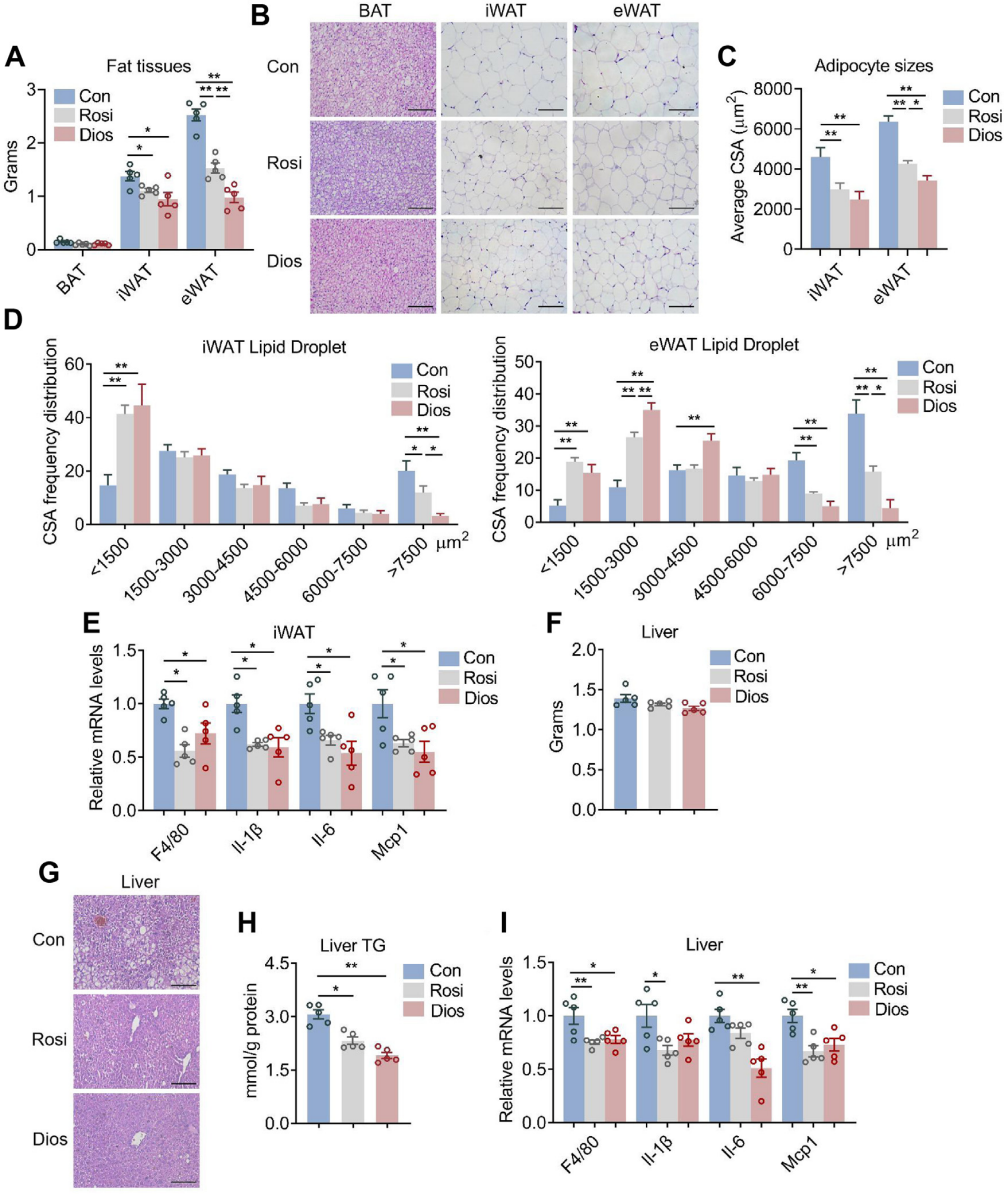
**Diosmin (Dios) ameliorates adipose tissue lipid accumulation and reduces hepatic steatosis of mice under high-fat diet (HFD).***A*–*I*, analysis of adipose tissues and liver in HFD-fed mice treated with control (Con), rosiglitazone (Rosi), or Dios (n = 5). *A*, tissue weights of brown adipose tissue (BAT), inguinal white adipose tissue (iWAT), and epididymal whote adipose tissue (eWAT) fat pads. *B*, representative images of H&E staining of fat tissues; The scale bar represents 100 μm. *C*, quantitative analysis of adipocyte sizes. *D*, cross-sectional area (CSA) frequency distribution of iWAT and eWAT. *E*, expression levels of inflammatory genes in iWAT. *F*, liver weights. *G*, representative H&E staining of liver. The scale bar represents 100 μm. *H*, liver triglyceride levels. *I*, expression levels of inflammatory genes in liver. Data are presented as mean ± SEM and ∗*p* < 0.05, ∗∗*p* < 0.01 compared with control group.

Next, we investigated the intrinsic alternation of metabolic tissues in diosmin-treated mice under HFD. Detailed analysis revealed that diosmin administration reduced adipose tissue weights with smaller adipocyte sizes in iWAT and epididymal WAT as shown by H&E staining and cross-sectional area quantification, as well as cross-sectional area frequency distribution (Fig. 6, *A*–*D*). Besides, inflammatory gene expressions were inhibited in iWAT of diosmin-treated mice under HFD, which was consistent with the role of PPAR in anti-inflammation (Fig. 6*E*).

Meanwhile, diosmin-treated mice showed decreased extent of liver steatosis as shown by reduced lipid infiltration and intrahepatic triglyceride levels, as well as suppressed inflammatory gene expressions compared with controls (Fig. 6, *F*–*I*). These data suggest that diosmin reduced adiposity and hepatic steatosis as well as ameliorating tissue inflammation in HFD.

Of note, although similarly to rosiglitazone treatment, local delivery of diosmin exhibited relative stronger effects in reducing adipose tissue weights and adipocyte sizes in iWAT and epididymal WAT (Fig. 6, *A*–*D*).

### Local diosmin administration in iWAT shows no obvious side effects in water retention and cardiovascular systems

**Figure 7:**
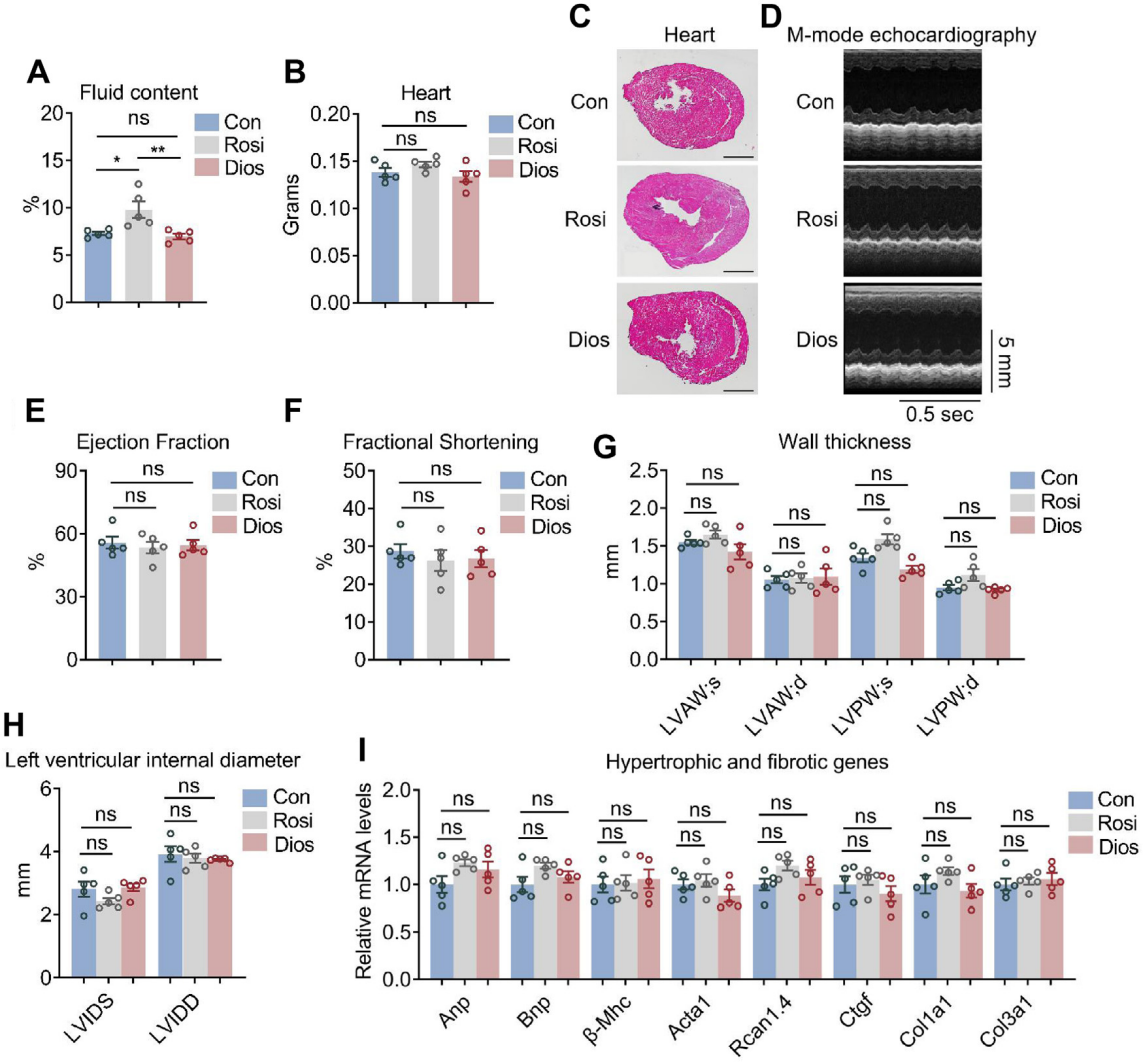
**Local diosmin (Dios) administration in inguinal white adipose tissue (iWAT) shows no obvious side effects in water retention and cardiovascular systems.***A*–*I*, analysis of fluid retention and cardiovascular functions in high-fat diet (HFD)-fed mice treated with control (Con), rosiglitazone (Rosi), or Dios (n = 5). *A*, fluid retention; (*B*) heart weights; (*C*) H&E staining of heart. The scale bar represents 10 μm. *D*, M-mode echocardiography. *E* and *F*, ejection fraction and fractional shortening. *G* and *H*, wall thickness (left ventricular anterior systolic/diastolic wall thickness (LVAW(s); LVAW(d)), left ventricular posterior systolic/diastolic wall thickness (LVPW(s); LVPW(d)), and left ventricular end systolic/diastolic diameter (LVIDS/LVIDD)). *I*, expression levels of hypertrophy and fibrotic genes in heart. Data are presented as mean ± SEM and ∗*p* < 0.05, ∗∗*p* < 0.01 compared with control group.

Oral PPARγ full-agonist treatment such as rosiglitazone causes an array of side effects, including abdominal fat accumulation, fluid retention, and increased risk of cardiac dysfunction (35). We found that local injection of rosiglitazone also caused increased fluid retention in mice as assessed by MRI, whereas in contrast, diosmin treatment showed no side effects on fluid retention (Fig. 7*A*). Moreover, serum parameter analysis showed no apparent toxicity effects upon diosmin treatment, whereas rosiglitazone treatment caused trends of increased serum urea nitrogen and creatinine levels that are indicative of increased kidney stresses (Table S1). Interestingly, local administration of diosmin and rosiglitazone did not lead to heart weight increase, heart muscle hypertrophy, or enlargement of cardiac cavity (Fig. 7, *B* and *C*). Consistently, M-mode echocardiography showed no obvious abnormalities in cardiac perimeters, that is, ejection fraction, fractional shortening (FS), wall thickness, left ventricular end systolic/diastolic diameter, as well as expressions of myocardial hypertrophy and fibrotic genes compared with control mice (Fig. 7, *D*–*I*), suggesting iWAT local delivery of diosmin or rosiglitazone did not cause cardiac overload.

Taken together, these data suggest that local administration of diosmin in iWAT significantly ameliorated insulin resistance, obesity, hyperlipidemia, and hepatic steatosis, without causing the classical adverse side effects of TZDs *in vivo*.

## Discussion

In the present study, we performed computer virtual screening from thousands of natural small molecules and their derivatives to look for potential PPARγ selective modulator that blocks phosphorylation of PPARγ at Ser273 site previously shown to exhibit metabolic benefits without apparent adverse effects. Molecular docking, chemical analysis, and molecular analysis revealed diosmin as a potential lead compound, which binds to PPARγ and blocks PPARγ Ser273 phosphorylation in beige adipocytes. Diosmin improves glucose uptake and diabetic gene sets dysregulated upon PPARγ S273 phosphorylation without altering adipogenic ability in beige adipocytes. *In vivo*, we found that diosmin improved insulin sensitivity as well as reduced body weight and enhanced energy expenditure in mice fed with HFD. Furthermore, diosmin promoted browning of iWAT and ameliorated hepatic steatosis with attenuated proinflammatory responses in iWAT and liver, with no apparent adverse effects such as edema or cardiovascular disease risk previously reported in PPARγ full agonist usage.

Diosmin is a natural compound obtained from hesperidin, a substance commonly found in citrus fruit and Rutaceae family, through semisynthesis (36, 37). In this study, we identified diosmin as a selective PPARγ modulator. PPARγ antagonist GW9662 or CDK5 inhibitor abolished the effects of diosmin, overall suggesting diosmin may function *via* PPARγ and its S273 phosphorylation. PPARγ has been shown to play an important role in vascular protection, whereas its activation suppresses the oxidative stress and apoptosis of endothelial cells, protects against vascular thrombosis, and alleviates atherosclerosis (38, 39, 40). Interestingly, diosmin also has a long history of clinical implication and has been widely used in the treatment of chronic venous disorders, including lymphatic drainage, microcirculation, venous tone, and capillary hyperpermeability clinically for its effectiveness and safeness (20, 41). Moreover, diosmin also has anti-inflammatory and antioxidant properties (42, 43). In clinical practices, flavonoid fraction containing 90% diosmin treatment scavenged oxygen free radicals in human neutrophils as well as decreased HbA1c level in type 1 diabetic patients (44). Besides, dietary supplement flebotrofine, containing diosmin improved the antioxidant and antithrombotic profile in both type 1 and type 2 diabetes (45). It is possible that these functions of diosmin and diosmin-containing substances may be exerted through its modulation on PPARγ. Future modifications based on diosmin as the lead compound may provide more attractive candidates against diabetes *via* blocking PPARγ S273 phosphorylation.

Beige fat has been considered as an attractive targeting tissue against metabolic diseases, considering its inducible activation in promoting energy metabolism and improving serum parameters as metabolic sink (46, 47). PRDM16 and PGC-1α form complex with PPARγ to regulate energy homeostasis. Of note, fat-specific PGC-1α deficiency develops insulin resistance and hyperlipidemia, and the effects were majorly caused by beige fat as shown by decreased expression of thermogenic and mitochondrial genes, whereas gene expression patterns in brown fat were not altered (48). In addition, adipocyte-specific deletion of PRDM16 inhibited beige adipocyte function in subcutaneous fat and promoted obesity, as well as aggravated insulin resistance and hepatic steatosis in mice under HFD, while caused minimal effects on brown fat (49). These findings indicate that beige fat plays an important role in metabolism. Considering the importance of PPARγ in beige fat functionality in concert with PGC-1α and PRDM16, as well as the unique role of beige fat in metabolism, we administrated diosmin locally in beige fat, instead of intraperitoneally or intravenously, to pursue a direct action on beige fat and metabolic improvement, while at the same time avoid any potential adverse effects *via* systemic administration. Further attempts of different delivery routes could be tested.

Notably, local injection of rosiglitazone caused increased fluid retention and serum kidney stress markers in mice, which side effect was absent in diosmin group, suggesting the existence of systemic exposure in mice injected with compound injection in iWAT. Interestingly, iWAT local delivery of both diosmin and rosiglitazone showed no overt cardiovascular defect including parameters in heart weights, ejection fraction, FS, wall thickness, left ventricular end systolic/diastolic diameter, as well as expressions of myocardial hypertrophy and fibrotic genes compared with control group. Overall, these data suggested that kidney and heart have different susceptibility toward compound exposure, and that different compounds have different impact on kidney. Systematic analysis of the pharmacokinetics in iWAT local compound delivery would be informative. In addition, acute oral delivery of rosiglitazone and diosmin both significantly promoted insulin sensitivity and brown gene programs in mice iWAT, whereas diosmin administration did not induce adipogenic genes as rosiglitazone did. Further analysis of the effects of long-term oral delivery of diosmin is warranted to systemically examine chronic metabolic changes. Overall, we have shown that diosmin has its unique features in treating obesity and metabolic derangement without overt adverse effect compared with rosiglitazone, thus may serve as a desirable alternative therapy for treating metabolic diseases.

PPARγ Ser273 phosphorylation has been shown to play a critical role in insulin resistance. Based on this feature, a series of compounds were developed to block PPARγ Ser273 phosphorylation and exerted hypoglycemic function without the usual adverse side effects of classical TZDs (35). Among these compounds, SR1664 and MRL24 showed no effects on reducing obesity, whereas we and others showed that in addition to improved glycemic control, diosmin, GQ-16, and roscovitine have additional effects on fat metabolism, including reducing body weight gain and hepatic steatosis of mice under HFD, possibly through enhanced formation of beige adipocytes in white adipose tissues and elevated energy expenditure (18, 19). It has been reported that PPARγ deacetylation (K268 and K293) *via* SIRT1 upon long-term TZD treatment protected against diet-induced obesity by enhancing browning of white adipose tissue and increasing energy expenditure, thus uncouples the adverse effects of TZD in weight gain from insulin sensitization (50, 51). Considering K268 and K293 locates closely to Ser273, those compounds, including diosmin, with antiobesity properties, may also block PPARγ acetylation and recruit the BAT program coactivator such as PRDM16, to selective induce BAT genes and repress visceral WAT genes associated with insulin resistance, which need further investigation.

Of note, the PPARγ partial agonists or nonagonist PPARγ ligands mentioned previously including SR1664, MRL24, GQ-16, and CDK5 inhibitor roscovitine have not been approved for clinical use. Meanwhile, a recent study showed that Gleevec, a well-known anticancer drug that exhibits dramatic effectiveness for the treatment of chronic myelocytic leukemia and gastrointestinal stromal tumors (52), also blocks the CDK5-mediated PPARγ S273 phosphorylation and improves insulin sensitivity without classical PPARγ agonism and related side effects. However, Gleevec is a very expensive anticancer drug, which limited its wide use for other milder but persisting diseases. Comparing with Gleevec, diosmin only costs 1/1000 of Gleevec in price and exhibited excellent antidiabetic effect, thus has great potential for the usage in patients with chronic metabolic diseases.

In conclusion, the present study reveals a previously unrecognized role of diosmin, a conventional drug features long-time clinical use and safeness, in improving insulin sensitivity by blocking PPARγ phosphorylation and reducing obesity without the side effects of classic PPARγ full agonists.

## Experimental procedures

### Virtual screening and molecular docking study

The crystal structure of human 7-bound PPARγ ligand-binding domain (Protein Data Bank code: 5GTO) was retrieved from the RCSB Protein Data Bank and imported into SYBYL software (Tripos) for the subsequent structural analysis and docking simulation. The ligand database consists of 3000 compounds from the Targetmol Bioactive Natural Compound Library (https://www.tsbiochem.com). After the protein and the ligand candidate preparation, molecular docking was carried out using the Surflex-Dock module of SYBYL with default settings, and “binding energy” was used as the indicator to choose the ligand with the lowest score, as well as target compounds were selected for further biological evaluation.

### Cell culture

Immortal beige preadipocytes were generously provided by Professor Qiurong Ding (Chinese Academy of Sciences) and cultured in Dulbecco’s modified Eagle's medium supplemented with 20% fetal bovine serum and 1% penicillin/streptomycin at 37 °C with 5% CO_2_. Beige adipocytes were differentiated as the following protocol. When preadipocytes reached confluency (day 0), cells were induced with 1 μg/ml insulin (catalog no.: HI0240; Eli Lilly), 0.5 mmol/l 3-isobutyl-1-methylxanthine (catalog no.: I7018, Sigma–Aldrich), 1 nmol/l T3 (catalog no.: T2877; Sigma–Aldrich), 1 μmol/l dexamethasone (catalog no.: D4902; Sigma–Aldrich) in the presence or the absence of 1 μmol/l rosiglitazone (catalog no.: R2408; Sigma–Aldrich). After 2 day differentiation, cells were switched to the maintenance medium containing 5 μg/ml insulin with or without 1 μmol/l rosiglitazone every 2 days. Differentiated beige adipocytes were treated with 0.3 mM palmitate (catalog no.: P0500; Sigma–Aldrich) for 48 h to mimic insulin resistance *in vitro*. For drug treatment, cells were treated with diosmin (catalog no.: D111390; Aladdin) instead of rosiglitazone for the whole differentiation process. GW9662 and roscovitine were purchased from MCE (catalog nos.: HY-30237 and HY-16578). Lipid accumulation in beige adipocytes treated with rosiglitazone or diosmin was detected by Oil Red O staining and measured at 520 nm by SpectraMax 190 microplate reader (Molecular Devices).

### Gene expression analyses

Total RNA was isolated from cultured cells or tissues by RNAiso Plus (catalog no.: 9109; TaKaRa), and 1 μg of total RNA was reversed transcribed into complementary DNA using the PrimeScript RT reagent Kit (catalog no.: PR047Q; TaKaRa). Quantitative real-time PCR was performed with the SYBR green fluorescent dye mix (catalog no.: 11143ES50; Yeasen) on the PCR system (LightCycle 480; Roche). Relative mRNA levels were calculated using the 2^−ΔΔ^Ct method, and sequences of primers used for real-time PCR were listed in Table S2.

### MST

The purified recombinant His-tagged PPAR protein was first labeled with the RED-tris-NTA second-generation dye. Diosmin and rosiglitazone were diluted from 2.5 mM and 10 M, respectively, and incubated with 200 nM labeled PPARγ at room temperature for 20 min in MST buffer (25 mM Tris–HCl, pH 8.0, 75 mM NaCl, 0.05% [v/v] Triton X-100). Then the samples were loaded into Monolith NT. Automated Premium Capillary Chip and analyzed *via* Monolith NT. Automated instrument. The data analysis was performed using MO Affinity Analysis (version 2.3).

### Cdk5 activity assay

Differentiated beige adipocytes were treated with rosiglitazone, diosmin, or roscovitine, and the cells were harvested and subjected to CDK5 activity assay using a CDK5/P25 kinase assay kit (catalog no.: HL50150; CG biotec). Total protein was measured using Enhanced BCA Protein Assay Kit (catalog no.: P0010S; Beyotime) as per the manufacturer's protocol.

### Immunoprecipitation and protein analyses

Differentiated beige adipocytes treated with rosiglitazone or diosmin were preincubated with TNF-α (50 ng/ml). Protein from cultured cells or tissues was extracted by the radioimmunoprecipitation assay buffer, and equal amounts of total protein were loaded into 10% acrylamide gels for general analysis and transferred to NC membranes (catalog no.: 66485; PALL). Membranes were incubated in 5% bovine serum albumin for 2 h and with primary antibodies overnight at 4 °C, including anti-p-PPARγ (1:2000 dilution) (catalog no.: bs-4888R; Bioss biotech), anti-PPARγ (1:1000 dilution) (catalog no.: sc-7273; Santa Cruz), anti-p-IRβ (1:2000 dilution) (catalog no.: 3025; Cell Signaling Technology), anti-p-AKT (1:2000 dilution) (catalog no.: 13038; Cell Signaling Technology), anti-p-GSK3β (1:2000 dilution) (catalog no.: 9322; Cell Signaling Technology) (1:2000 dilution), anti-UCP1 (catalog no.: Ab10983; Abcam), or β-actin (1:2000 dilution) (catalog no.: sc-47778; Santa Biotechnology). After washed three times with PBS with Tween-20, the corresponding horseradish peroxidase–conjugated secondary antibodies were then incubated for 2 h at room temperature. Detection was performed using Odyssey CLx Imaging System (LI-COR).

### Reporter gene assay

Human embryonic kidney 293 cells were transfected with PPRE luciferase reporter plasmid, PPARγ, RXRα, and Renilla using Lipofectamine 2000 (catalog no.: 11668019; Invitrogen). Then the cells were treated with rosiglitazone or diosmin for 24 h after an overnight transfection. The cells were harvested and performed with the reporter gene assay using a Double-luciferase reporter assay kit (catalog no.: FR201; Transgen). Luciferase activity was normalized to renillia activity.

### Glucose uptake assay

The beige adipocytes treated with or without diosmin were washed twice by PBS and maintained in fetal bovine serum–free medium for 2 h. After washing twice by PBS, cells were incubated with insulin for 0.5 h at 37 °C followed by the stock solution of radioactive 2-DG, which was added into each well for 0.5 h at 37 °C. The uptake was terminated by washing the cells with precooling PBS, and 300 μl of 0.05 M NaOH was added into each well for lysing cells. The entire cell lysate was transferred to a scintillation vial containing 3 ml liquid scintillation cocktail. Quantify the radioactivity associated with the cells using a liquid scintillation counter.

### Animal studies

Eight-week-old male C57BL/6J mice at around 22 to 25 g were purchased from Shanghai Research Center for Model Organisms and fed with a 12:12-h light/dark cycle with free access to food and water. All efforts were made to minimize animal suffering. For acute iWAT local drug treatment, mice were injected with 10 mg/kg diosmin, hesperidin, polydatin, amygdalin, or rosiglitazone in one side of the inguinal fat pads and injected with solvent control in the other side of inguinal fat pads in C57BL/6J mice and were sacrificed after 3 days. For acute oral delivery, 10 mg/kg rosiglitazone, diosmin, or solvent control was gavaged in C57BL/6J mice and sacrificed after 3 days. For chronic drug treatment, mice under HFD (catalog no.: D12492; Research Diet) were bilaterally injected in inguinal fat pads with 10 mg/kg diosmin, rosiglitazone, or solvent control in mice on Monday and Thursday of each week for 12 weeks. The body weight and food intake were monitored weekly. Body fat content was determined every 2 weeks by AccuFat-1050 MRI system (Meg-Med). After 12-week treatment, metabolic parameters were analyzed. For acute cold exposure, mice were individually caged without bedding and exposed to 4 °C for 6 h. Rectal temperature was measured each hour (Braintree). For determination of whole-body energy expenditure and basal metabolic rate of mice, oxygen consumption (VO_2_), carbon dioxide production (VCO_2_), and physical activity were measured using Comprehensive Lab Animal Monitoring System (Columbus Instruments) for 72 h. For the glucose tolerance test, mice were fasted for 16 h and injected with d-glucose in saline solution (1.5 g/kg) intraperitoneally, and plasma glucose levels were measured at 0, 15, 30, 60, 90, and 120 min (AlphaTrak Blood Glucose Monitor System; Abbott). For the insulin tolerance test, mice were injected with insulin in saline solution (1.25 U/kg body weight) intraperitoneally, and plasma glucose levels were measured at 0, 15, 30, 60, 90, and 120 min. Fluid retention was determined by AccuFat-1050 MRI system (Meg-Med). All animal studies were carried out following the guidelines approved by the Ethics Committee of Animal Experiments of East China Normal University (m20200604).

### Histological and immunohistochemistry analyses

Adipose and liver tissues were dissected and fixed in 10% neutral formalin, whereas heart tissues were fixed in 4% paraformaldehyde. All these samples were dehydrated and embedded in molten paraffin wax, and then paraffin blocks were cut into 5 mm sections and were subjected to hematoxylin and eosin staining. Sections were examined by light microscopy (Abaton Scan 300/Color scanner).

### Serum parameter and liver triglyceride level determination

Serum parameters including total cholesterol, high-density lipoprotein cholesterol and low-density lipoprotein cholesterol levels were measured with the commercial kits (A111-2, A112-2, and A113-2; Jiancheng). Serum chemistry tests including alanine aminotransferase, aspartate aminotransferase, creatine kinase, lactate dehydrogenase, urea nitrogen, creatinine, uric acid, albumin, and total protein were performed with the commercial kits (MAK052, MAK055, MAK116, MAK066, MAK006, MAK080, MAK077; 09753 and 71285-M; Sigma–Aldrich). Triglyceride was extracted using 5% NP-40 solution in liver and heated at 90 °C for twice. Then it was cooled down to room temperature and centrifuged at 12,000 rpm for 2 min to collect transparent supernatant. The serum and hepatic levels of triglyceride were measured using TG kit (K952; BioVision).

### Echocardiography

Mice were anesthetized by 1.5 to 2% isoflurane and reduced to 0.5 to 1% once the mouse was asleep. Oxygen gas was flowing at 2 ml/min. The chest skin of the mouse was shaved using a hair remover, and the heart function was evaluated with a 30 MHz high-frequency ultrasound transducer (Visualsonics; VEVO 2100). 2D image and M-mode echocardiographic images were studied in the parasternal short-axis view at the level of the papillary muscles. Left ventricular end-diastolic diameter and left ventricular end-systolic dimension (LVESD) were measured, and FS was calculated as follows: FS% = ([LVEDD − LVESD]/LVEDD) × 100%.

### Statistical analyses

Two-tailed unpaired Student’s *t* test and two-way ANOVA were performed to evaluate statistical significance using GraphPad Prism software (GraphPad Software, Inc). The *p* values were designed as follows: ∗*p* < 0.05 and ∗∗*p* < 0.01. All values specified in the figures were represented as mean ± SEM.

## Data availability

All data are contained within the article and supporting information.

## Supporting information

This article contains supporting information.

## Conflict of interest

The authors declare that they have no conflicts of interest with the contents of this article.

## Supporting information

Supplemental Tables S1 and S2

Supplemental Figure S1 **Transcriptional activity of PPARγ under Hesperidin, Polydatin or Amygdalin treatment.***A*, transcriptional activity of a PPARγ-responsive element (PPRE) after treatment with Hesperidin, Polydatin and Amygdalin (n = 3). Data are presented as mean ± SEM and ∗*p* < 0.05, ∗∗*p* < 0.01 compared to control group.

Supplemental Figure S2 **Acute Hesperidin, Polydatin and Amygdalin iWAT local administration did not show effects on diabetic gene programs.** Mice were injected with 10 mg/kg Rosiglitazone (Rosi), Hesperidin (Hesp), Polydatin (Poly) or Amygdalin (Amyg) into one side of inguinal fat pads and injected with solvent control (Con) in the other side of inguinal fat pads and sacrificed after 3 days and analyze for (*A*) Expression of gene sets regulated by PPARγ S273 phosphorylation in iWAT local treated with control, Rosiglitazone, Hesperidin, Polydatin and Amygdalin (n = 4). *B*, protein levels of S273 p-PPARγ, (*C*) p-IRβ, p-AKT and p-GSK3β in iWAT of mice. Data are presented as mean ± SEM and ∗*p* < 0.05, ∗∗*p* < 0.01 compared to control group.

Supplemental Figure S3 **Acute Diosmin oral administration improves diabetic gene programs in iWAT of mice.***A*, experimental model of acute oral gavage of control (Con), Rosiglitazone (Rosi) or Diosmin (Dios) in mice at 10 mg/kg and sacrificed after 3 days. (n = 5). *B*, protein levels of S273 p-PPARγ, (*C*) p-IRβ, p-AKT and p-GSK3β, (*D*) expression of gene set regulated by PPARγ S273 phosphorylation in iWAT of mice after acute Diosmin or Rosiglitazone administration. *E*, expression levels of brown gene programs, adipogenic marker genes, (*F*) UCP1 protein levels in iWAT of mice after acute Diosmin or Rosiglitazone administration. Data are presented as mean ± SEM and ∗*p* < 0.05, ∗∗*p* < 0.01 compared to control group.

Supplemental Figure S4 **Diosmin improves glucose metabolism in mice under HFD.***A* and *B*, the fasting and random glucose levels of HFD fed mice treated with control (Con), Rosiglitazone (Rosi) or Diosmin (Dios) (n = 5). Data are presented as mean ± SEM and ∗*p* < 0.05, ∗∗*p* < 0.01 compared to control group.
