# Supplementary material for: Selective PPARγ modulator diosmin improves insulin sensitivity and promotes browning of white fat
Source: J Biol Chem. 2023 Feb 24;299(4):103059. doi: 10.1016/j.jbc.2023.103059 (PMC10033317; doi:10.1016/j.jbc.2023.103059)
Supplement: Supplemental Tables S1 and S2 [file mmc1.docx]

**Table S1. Serum chemistry tests of high fat diet fed mice treated with control, Rosiglitazone or Diosmin (n=5 per group).**

| **Tests** | **HFD** | **HFD+**  **Rosiglitazone** | ***P*** | **HFD+**  **Diosmin** | ***P*** |
| --- | --- | --- | --- | --- | --- |
| Urea nitrogen (mmol/L) | 13.04±2.14 | 15.14±0.82 | 0.10 | 13.4±1.77 | 0.81 |
| Creatinine (μmol/L) | 28.41±5.27 | 33.34±2.74 | 0.14 | 24.28±8.02 | 0.41 |
| Uric acid (μmol/L) | 189.95±20.50 | 187.30±15.09 | 0.84 | 196.26±22.49 | 0.69 |
| Albumin (g/L) | 40.66±3.48 | 38.27±3.05 | 0.33 | 38.03±2.54 | 0.26 |
| Total protein (g/L) | 25.64±1.12 | 25.98±1.23 | 0.69 | 25.52±1.10 | 0.88 |
| Alanine aminotransferase (U/L) | 39.01±9.73 | 33.72±4.26 | 0.35 | 30.1±10.07 | 0.24 |
| Aspartate aminotransferase (U/L) | 104.5±15.89 | 95.03±10.59 | 0.35 | 92.52±15.25 | 0.31 |
| Creatine kinase (U/L) | 877.91±36.72 | 847.22±31.76 | 0.24 | 849.61±50.74 | 0.39 |
| Lactate dehydrogenase (U/L) | 297.49±23.38 | 294.37±12.18 | 0.82 | 291.96±19.26 | 0.72 |

**Table S2. qPCR primers used in the study**

| Gene name | Primer sequence (5′→3′) | |
| --- | --- | --- |
| m36B4 | Forward | AGATTCGGGATATGCTGTTGGC |
|  | Reverse | TCGGGTCCTAGACCAGTGTTC |
| mUcp1 | Forward | GGCCCTTGTAAACAACAAAATAC |
|  | Reverse | GGCAACAAGAGCTGACAGTAAAT |
| mCidea | Forward | TGACATTCATGGGATTGCAGAC |
|  | Reverse | CGAGCTGGATGTATGAGGGG |
| mElovl3 | Forward | TTCTCACGCGGGTTAAAAATGG |
|  | Reverse | TCTCGAAGTCATAGGGTTGCAT |
| mPrdm16 | Forward | CCACCAGCGAGGACTTCAC |
|  | Reverse | GGAGGACTCTCGTAGCTCGAA |
| mAtpaseβ | Forward | GACATGGGCACAATGCAGG |
|  | Reverse | GCAGGGTCAGTCAGGTCATCA |
| mCytc | Forward | AAATCTCCACGGTCTGTTCGG |
|  | Reverse | GGGTATCCTCTCCCCAGGTG |
| mPparγ | Forward | TCGCTGATGCACTGCCTATG |
|  | Reverse | GAGAGGTCCACAGAGCTGATT |
| mCd36 | Forward | TTTGGAGTGGTAGTAAAAAGGGC |
|  | Reverse | TGACATCAGGGACTCAGAGTAG |
| mAp2 | Forward | ACACCGAGATTTCCTTCAAACTG |
|  | Reverse | CCATCTAGGGTTATGATGCTCTTC |
| mCyp2f2 | Forward | GTCGGTGTTCACGGTGTACC |
|  | Reverse | AAAGTTCCGCAGGATTTGGAC |
| mRarres2 | Forward | GCCTGGCCTGCATTAAAATGG |
|  | Reverse | CTTGCTTCAGAATTGGGCAGT |
| mSelenbp1 | Forward | ATGGCTACAAAATGCACAAAGTG |
|  | Reverse | CCTGTGTTCCGGTAAATGCAG |
| mCar3 | Forward | TGACAGGTCTATGCTGAGGGG |
|  | Reverse | CAGCGTATTTTACTCCGTCCAC |
| mPeg10 | Forward | TGCTTGCACAGAGCTACAGTC |
|  | Reverse | AGTTTGGGATAGGGGCTGCT |
| mCidec | Forward | ATGGACTACGCCATGAAGTCT |
|  | Reverse | CGGTGCTAACACGACAGGG |
| mCd24a | Forward | GTTGCACCGTTTCCCGGTAA |
|  | Reverse | CCCCTCTGGTGGTAGCGTTA |
| mAcyl | Forward | CAGCCAAGGCAATTTCAGAGC |
|  | Reverse | CTCGACGTTTGATTAACTGGTCT |
| mNr1d2 | Forward | TGAACGCAGGAGGTGTGATTG |
|  | Reverse | GAGGACTGGAAGCTATTCTCAGA |
| mNr3c1 | Forward | AGCTCCCCCTGGTAGAGAC |
|  | Reverse | GGTGAAGACGCAGAAACCTTG |
| mRybp | Forward | CGACCAGGCCAAAAAGACAAG |
|  | Reverse | CACATCGCAGATGCTGCATT |
| mTxnip | Forward | TCTTTTGAGGTGGTCTTCAACG |
|  | Reverse | GCTTTGACTCGGGTAACTTCACA |
| mNr1d1 | Forward | TACATTGGCTCTAGTGGCTCC |
|  | Reverse | CAGTAGGTGATGGTGGGAAGTA |
| mAdiponectin | Forward | TGTTCCTCTTAATCCTGCCCA |
|  | Reverse | CCAACCTGCACAAGTTCCCTT |
| mAdipsin | Forward | CATGCTCGGCCCTACATGG |
|  | Reverse | CACAGAGTCGTCATCCGTCAC |
| mDdx17 | Forward | TCTTCAGCCAACAATCCCAATC |
|  | Reverse | GGCTCTATCGGTTTCACTACG |
| mAplp2 | Forward | GTGGTGGAAGACCGTGACTAC |
|  | Reverse | TCGGGGGAACTTTAACATCGT |
| mF4/80 | Forward | CTGAGGATGAATTCCCGTGT |
|  | Reverse | GTCTCGGATGCTTCCACAAT |
| mIl-1β | Forward | AAATACCTGTGGCCTTGGGC |
|  | Reverse | CTTGGGATCCACACTCTCCAG |
| mIl-6 | Forward | TAGTCCTTCCTACCCCAATTTCC |
|  | Reverse | TTGGTCCTTAGCCACTCCTTC |
| mMcp1 | Forward | TTAAAAACCTGGATCGGAACCAA |
|  | Reverse | GCATTAGCTTCAGATTTACGGGT |
| mAnp | Forward | TCTTCCTCGTCTTGGCCTTT |
|  | Reverse | CCAGGTGGTCTAGCAGGTTC |
| mBnp | Forward | TGGGAGGTCACTCCTATCCT |
|  | Reverse | GGCCATTTCCTCCGACTTT |
| mβ-Mhc | Forward | CGGACCTTGGAAGACCAGAT |
|  | Reverse | GACAGCTCCCCATTCTCTGT |
| mActa1 | Forward | CCAAAGCTAACCGGGAGAAG |
|  | Reverse | GACAGCACCGCCTGGATAG |
| mRcan1.4 | Forward | TTGTGTGGCAAACGATGATGT |
|  | Reverse | CCCAGGAACTCGGTCTTGT |
| mCtgf | Forward | CTTCTGCAGACTGGAGAAGC |
|  | Reverse | CAGCCAGAAAGCTCAAACTTG |
| mCol1a1 | Forward | CTGGCGGTTCAGGTCCAAT |
|  | Reverse | TTCCAGGCAATCCACGAGC |
| mCol3a1 | Forward | TGAATGGTGGTTTTCAGTTCAG |
|  | Reverse | GATCCCATCAGCTTCAGAGACT |
