# Supplementary figures and images for: Selective PPARγ modulator diosmin improves insulin sensitivity and promotes browning of white fat
Source: J Biol Chem. 2023 Feb 24;299(4):103059. doi: 10.1016/j.jbc.2023.103059 (PMC10033317; doi:10.1016/j.jbc.2023.103059)

**Figure S1**

**A**

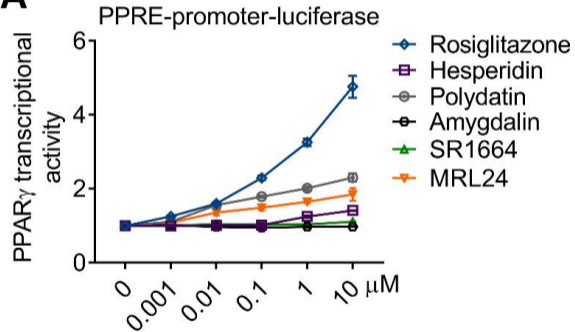

Supplement: Supplemental Figure S1 — Transcriptional activity of PPARγ under Hesperidin, Polydatin or Amygdalin treatment.A, transcriptional activity of a PPARγ-responsive element (PPRE) after treatment with Hesperidin, Polydatin and Amygdalin (n = 3). Data are presented as mean ± SEM and ∗p < 0.05, ∗∗p < 0.01 compared to control group. [file mmc2.pdf]

**Figure S2**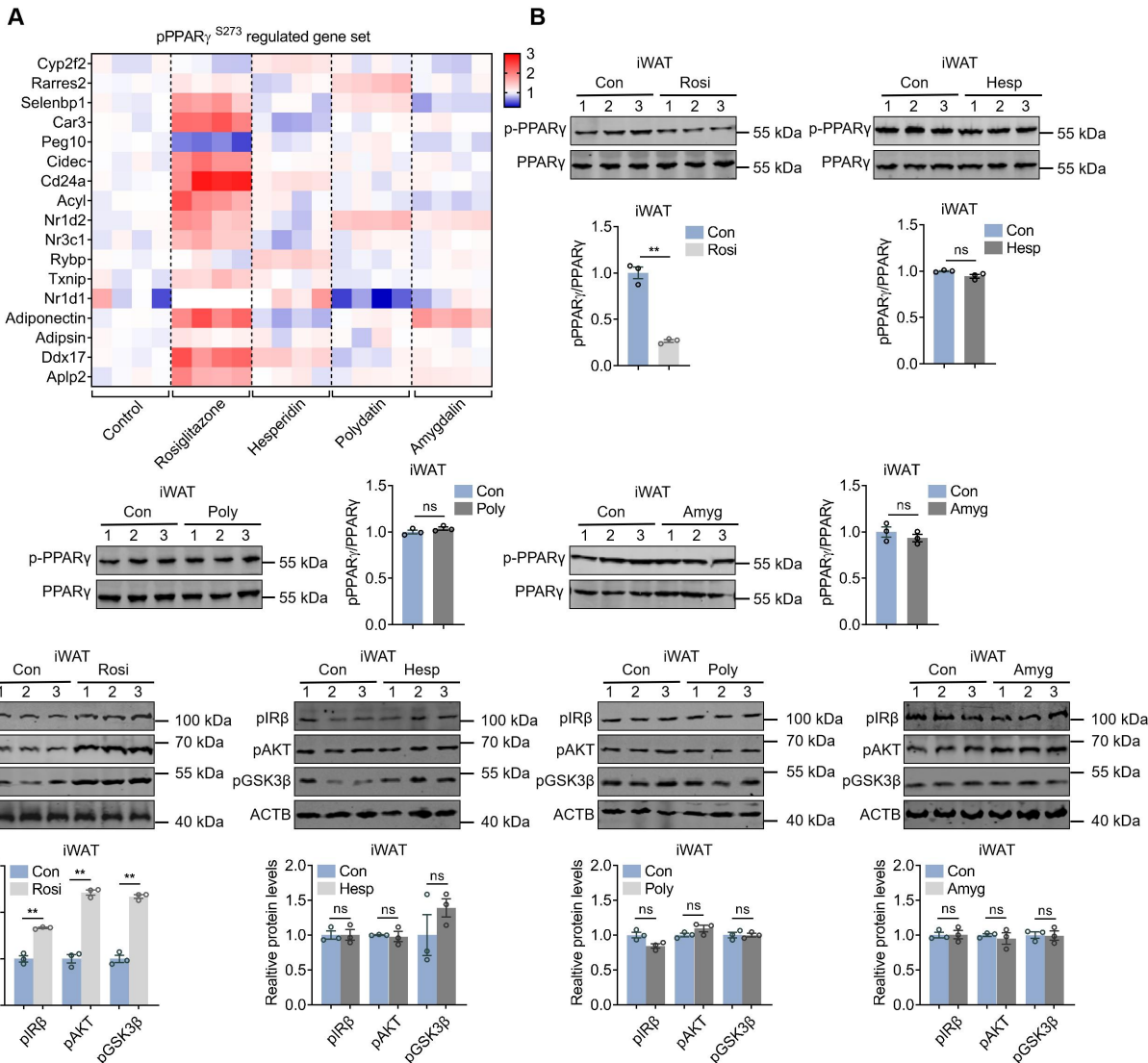

Supplement: Supplemental Figure S2 — Acute Hesperidin, Polydatin and Amygdalin iWAT local administration did not show effects on diabetic gene programs. Mice were injected with 10 mg/kg Rosiglitazone (Rosi), Hesperidin (Hesp), Polydatin (Poly) or Amygdalin (Amyg) into one side of inguinal fat pads and injected with solvent control (Con) in the other side of inguinal fat pads and sacrificed after 3 days and analyze for (A) Expression of gene sets regulated by PPARγ S273 phosphorylation in iWAT local treated with control, Rosiglitazone, Hesperidin, Polydatin and Amygdalin (n = 4). B, protein levels of S273 p-PPARγ, (C) p-IRβ, p-AKT and p-GSK3β in iWAT of mice. Data are presented as mean ± SEM and ∗p < 0.05, ∗∗p < 0.01 compared to control group. [file mmc3.pdf]

Figure S3

A

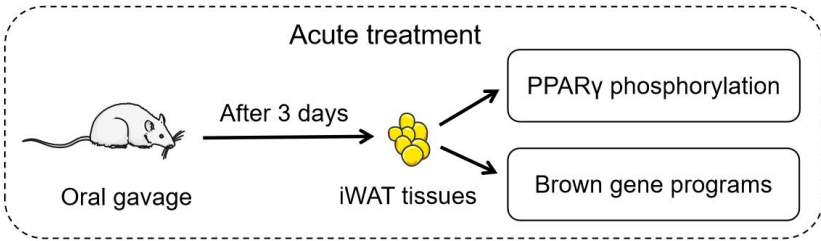

B

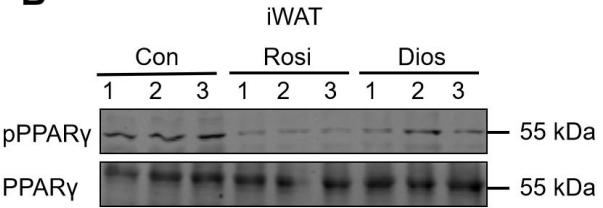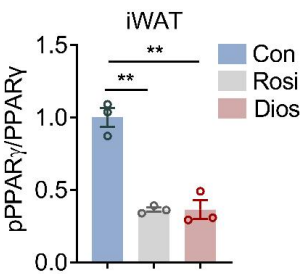

C

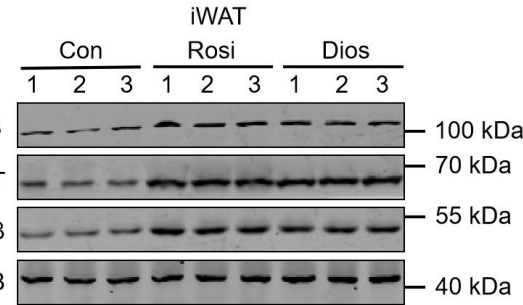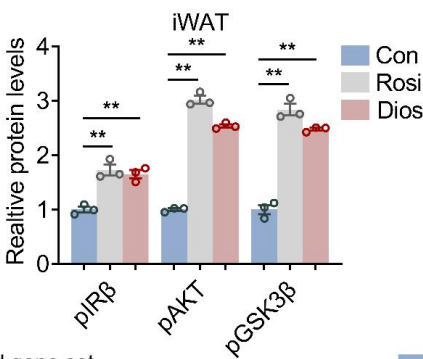

D

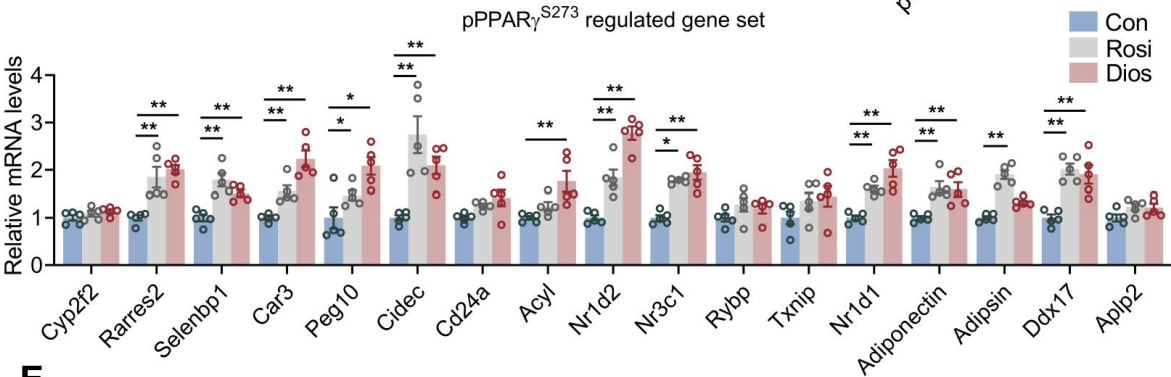

E

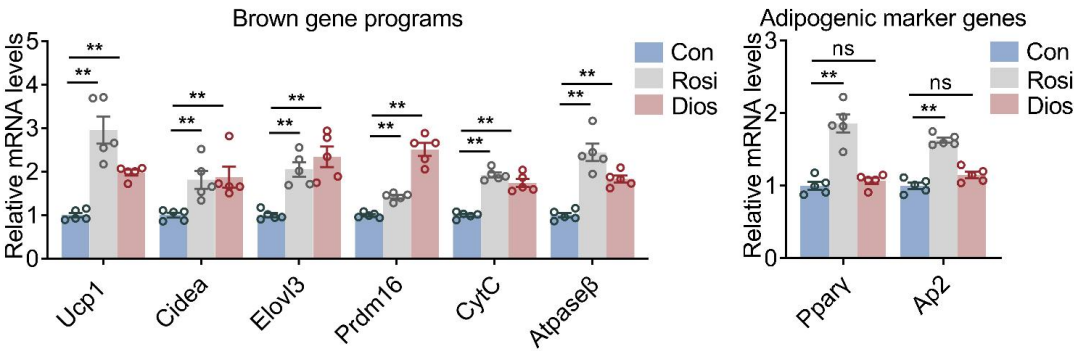

F

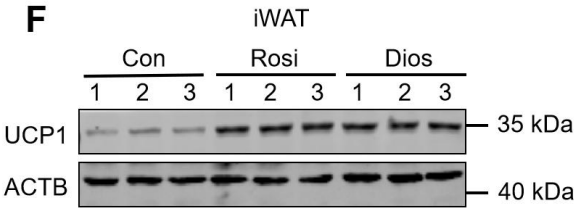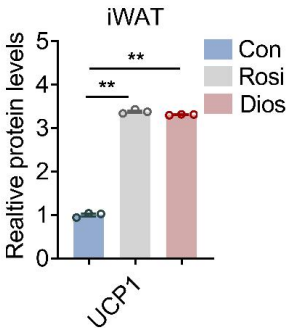

Supplement: Supplemental Figure S3 — Acute Diosmin oral administration improves diabetic gene programs in iWAT of mice.A, experimental model of acute oral gavage of control (Con), Rosiglitazone (Rosi) or Diosmin (Dios) in mice at 10 mg/kg and sacrificed after 3 days. (n = 5). B, protein levels of S273 p-PPARγ, (C) p-IRβ, p-AKT and p-GSK3β, (D) expression of gene set regulated by PPARγ S273 phosphorylation in iWAT of mice after acute Diosmin or Rosiglitazone administration. E, expression levels of brown gene programs, adipogenic marker genes, (F) UCP1 protein levels in iWAT of mice after acute Diosmin or Rosiglitazone administration. Data are presented as mean ± SEM and ∗p < 0.05, ∗∗p < 0.01 compared to control group. [file mmc4.pdf]

# Figure S4

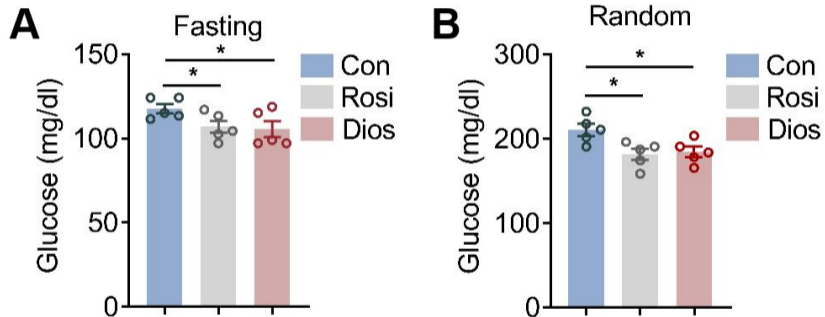

Supplement: Supplemental Figure S4 — Diosmin improves glucose metabolism in mice under HFD.A and B, the fasting and random glucose levels of HFD fed mice treated with control (Con), Rosiglitazone (Rosi) or Diosmin (Dios) (n = 5). Data are presented as mean ± SEM and ∗p < 0.05, ∗∗p < 0.01 compared to control group. [file mmc5.pdf]
